# Controlled Administration of Penicillamine Reduces Radiation Exposure in Critical Organs during ^64^Cu-ATSM Internal Radiotherapy: A Novel Strategy for Liver Protection

**DOI:** 10.1371/journal.pone.0086996

**Published:** 2014-01-22

**Authors:** Yukie Yoshii, Hiroki Matsumoto, Mitsuyoshi Yoshimoto, Takako Furukawa, Yukie Morokoshi, Chizuru Sogawa, Ming-Rong Zhang, Hidekatsu Wakizaka, Hiroshi Yoshii, Yasuhisa Fujibayashi, Tsuneo Saga

**Affiliations:** 1 Molecular Imaging Center, National Institute of Radiological Sciences, Chiba, Japan; 2 Research Centre, Nihon Medi-Physics Co., Ltd., Chiba, Japan; 3 Division of Functional Imaging, National Cancer Center Hospital East, Chiba, Japan; 4 Emergency Radiation Exposure Medical Care Research Center, National Institute of Radiological Sciences, Chiba, Japan; University of California Davis, United States of America

## Abstract

**Purpose:**

^64^Cu-diacetyl-bis (*N* ^4^-methylthiosemicarbazone) (^64^Cu-ATSM) is a promising theranostic agent that targets hypoxic regions in tumors related to malignant characteristics. Its diagnostic usefulness has been recognized in clinical studies. Internal radiotherapy (IRT) with ^64^Cu-ATSM is reportedly effective in preclinical studies; however, for clinical applications, improvements to reduce radiation exposure in non-target organs, particularly the liver, are required. We developed a strategy to reduce radiation doses to critical organs while preserving tumor radiation doses by controlled administration of copper chelator penicillamine during ^64^Cu-ATSM IRT.

**Methods:**

Biodistribution was evaluated in HT-29 tumor-bearing mice injected with ^64^Cu-ATSM (185 kBq) with or without oral penicillamine administration. The appropriate injection interval between ^64^Cu-ATSM and penicillamine was determined. Then, the optimal penicillamine administration schedule was selected from single (100, 300, and 500 mg/kg) and fractionated doses (100 mg/kg×3 at 1- or 2-h intervals from 1 h after ^64^Cu-ATSM injection). PET imaging was performed to confirm the effect of penicillamine with a therapeutic ^64^Cu-ATSM dose (37 MBq). Dosimetry analysis was performed to estimate human absorbed doses.

**Results:**

Penicillamine reduced ^64^Cu accumulation in the liver and small intestine. Tumor uptake was not affected by penicillamine administration at 1 h after ^64^Cu-ATSM injection, when radioactivity was almost cleared from the blood and tumor uptake had plateaued. Of the single doses, 300 mg/kg was most effective. Fractionated administration at 2-h intervals further decreased liver accumulation at later time points. PET indicated that penicillamine acts similarly with the therapeutic ^64^Cu-ATSM dose. Dosimetry demonstrated that appropriately scheduled penicillamine administration reduced radiation doses to critical organs (liver, ovaries, and red marrow) below tolerance levels. Laxatives reduced radiation doses to the large intestine.

**Conclusions:**

We developed a novel strategy to reduce radiation exposure in critical organs during ^64^Cu-ATSM IRT, thus promoting its clinical applications. This method could be beneficial for other ^64^Cu-labeled compounds.

## Introduction

^64^Cu-diacetyl-bis (*N* ^4^-methylthiosemicarbazone) (^64^Cu-ATSM) is a promising theranostic agent that targets hypoxic regions in tumors [1], [2], [3]. Radiolabeled Cu-ATSM was originally developed as a positron emission tomography (PET) imaging agent for hypoxia [2], [4], [5], [6], [7]. Clinical PET studies with Cu-ATSM have been already performed, and the usefulness of this agent has been recognized; for example, Cu-ATSM uptake correlated with therapeutic resistance and metastatic potential in several tumors, including uterine cervical carcinoma and rectal carcinoma [4], [7], [8]. The mechanism of radiolabeled Cu-ATSM accumulation in hypoxic regions of tumor tissues has been well studied. Cu-ATSM has a high membrane permeability and thus rapidly diffuses into cells and is reduced and trapped within cells under highly reduced intracellular conditions such as hypoxia [5], [9], [10], [11], [12]. Cu-ATSM uptake also reportedly reflects the level of the biological reductant NAD(P)H, which is associated with hypoxia or mitochondrial dysfunction, and the activity of NAD(P)H-dependent reductive enzymes, rather than oxygenic conditions directly [10], [13], [14], [15]. From these results, Cu-ATSM is considered a surrogate marker of tumor hypoxia via the detection of an intracellular over-reduced status.

Among the available Cu radioisotopes (^60^Cu, ^61^Cu, ^62^Cu, ^64^Cu, and ^67^Cu), ^64^Cu has several advantages for clinical applications because of its unique properties. First, ^64^Cu decays via β^+^ (0.653 MeV, 17.4%), β^−^ (0.574 MeV, 40%), and electron capture (42.6%); thus, γ-ray photons from electron-positron annihilation can be detected by PET, while β^−^ particle and Auger electrons emitted from this nuclide can damage tumor cells [1]. Therefore, ^64^Cu-ATSM could be used as a theranostic agent, which would enable PET-guided therapy for various tumors [1], [2], [3]. Second, ^64^Cu can be readily produced with a small on-site cyclotron. Due to its relatively long half-life (t_1/2_ = 12.7 h) among PET radioisotopes, the ^64^Cu delivery is also feasible. In fact, ^64^Cu delivery has already begun in the United States, and a multicenter clinical trial of ^64^Cu-ATSM has been conducted, although this agent is still limited to diagnostic purposes [8].

The therapeutic effects of ^64^Cu-ATSM have also been demonstrated in preclinical studies [1], [3], [16], [17]. ^64^Cu-ATSM has been reported to reduce the clonogenic survival of tumor cells and induce post-mitotic apoptosis in vitro [3]. Lewis et al. have demonstrated the feasibility of ^64^Cu-ATSM as a radiotherapeutic agent in detail, using tumor-bearing hamsters, and have shown that ^64^Cu-ATSM treatment increased survival time with no serious toxicity [1]. Furthermore, we recently reported that, in the Colon-26 tumor-bearing mouse model, ^64^Cu-ATSM preferentially localized in intratumoral regions with high densities of CD133^+^ cells, which are designated as cancer stem cells or cancer stem cell-like cells. Additionally, ^64^Cu-ATSM IRT inhibited tumor growth and killed not only CD133^−^ cancer cells, but also CD133^+^ cells [17], [18]. CD133^+^ cells are reportedly radiotherapy/chemotherapy resistant and possess a high metastatic potential [19], [20]. These findings indicated that ^64^Cu-ATSM has great potential not only as a diagnostic tool, but also as a therapeutic option for patients and that ^64^Cu-ATSM IRT might be a promising therapeutic approach for targeting tumor malignant characteristics such as tumor hypoxia and cancer stem cell-like cells.

However, when considering the clinical applications of ^64^Cu-ATSM for therapeutic use, the high physiological accumulation of ^64^Cu in non-target organs is a major barrier. In a clinical PET study with Cu-ATSM, the liver was reported to be the principal dose-limiting organ in humans [21]. In a hamster model, ^64^Cu accumulation was high in the intestines, as well as in the liver [1]. Hence, the reduction of radiation exposure in these organs, especially the liver, is important to minimize adverse effects and facilitate the clinical application of ^64^Cu-ATSM IRT.

We focused on the heavy-metal chelator penicillamine to develop an effective method for reducing radiation absorption doses in critical organs. Penicillamine is an active ingredient of drugs used to treat intoxication by heavy metals such as Cu, Hg, Zn, and Pb, and penicillamine treatment enhances the urinary excretion of these metals [22], [23]. Penicillamine has also been approved for the treatment of Wilson’s disease, a genetic copper metabolism disorder. Copper is essential to the physiological functions of a number of enzymes such as tyrosinase, cytochrome oxidase, and superoxide dismutase; however, poorly regulated Cu levels in body fluids can be toxic [24]. Under normal conditions, Cu homeostasis is maintained through enterohepatic circulation, and excess Cu is excreted into the bile and feces. In Wilson’s disease, Cu transport to the bile is disrupted by a mutation in a Cu-transporting P-type ATPase, ATP7B, which leads to an abnormal accumulation of Cu in the liver and subsequently results in hepatic cirrhosis, cardiac dysfunction, pancreatic dysfunction, and neurological abnormalities [25]. The action mechanism of penicillamine in Wilson’s disease has been reported; this agent binds Cu in the small intestine and enhances urinary Cu excretion, resulting in the prevention of Cu enterohepatic circulation and reduced Cu levels in the liver [24]. Additionally, another study of radiolabeled penicillamine showed that this drug, when administered orally (p.o.), is delivered to the liver [26], where it will remove accumulated Cu. Accordingly, we hypothesized that penicillamine could reduce ^64^Cu accumulation in the liver and small intestine and possibly other organs after ^64^Cu-ATSM administration. In this study, we developed a method to reduce ^64^Cu accumulation in non-target organs but not in tumors by appropriately scheduling the administration of penicillamine. The effect of the combined use of laxatives and penicillamine on reduced ^64^Cu accumulation, particularly in the large intestine, was also tested.

## Materials and Methods

### Ethics Statement

Animal experiments in this study were performed in strict accordance with the recommendations in the Guide for the Care and Use of Laboratory Animals of the National Institute of Radiological Sciences (Japan). The protocol was approved by the Animal Ethics Committee of the National Institute of Radiological Sciences (Japan; Permit Number: M40-01). All efforts were made to minimize suffering during the animal experiments.

### Preparation of ^64^Cu-ATSM and Penicillamine

^64^Cu was produced as reported previously [27]. The purification of ^64^Cu and the preparation of ^64^Cu-ATSM were performed according to previously reported procedures [18], [27]. The radiochemical purity of the resulting ^64^Cu-ATSM was greater than 95%, as determined by silica gel thin-layer chromatography (TLC; silica gel 60; Merck, Whitehouse Station, NJ, USA) with ethyl acetate as the mobile phase [28]. Radioactivity levels on the TLC plates were analyzed with a bioimaging analyzer (FLA-7000; Fujifilm, Tokyo, Japan). Penicillamine was obtained from the Tokyo Chemical Industry (Tokyo, Japan).

### Penicillamine Challenge to ^64^Cu-ATSM in Mouse Plasma

The trans-chelation of ^64^Cu from ^64^Cu-ATSM to penicillamine was investigated in mouse plasma. For this study, ^64^Cu-ATSM (370 MBq/mL) and penicillamine (83 mg/mL) solutions were prepared in saline. Freshly prepared mouse plasma was pre-incubated at 37°C for 10 min. The ^64^Cu-ATSM solution, penicillamine solution or saline, and plasma were mixed together in a volume ratio of 10∶15:100, and the mixture was incubated at 37°C. Aliquots were removed at 0, 5, 10, and 30 min after the start of the incubation and were subsequently loaded onto TLC plates. Radioactivity on the TLC plates was analyzed using a bioimaging analyzer, and the relative ratios of the activity were calculated for the fraction with intact ^64^Cu-ATSM (Rf = 0.8) and the fraction with free ^64^Cu and ^64^Cu-penicillamine at the origin to the total activity.

### Cell Line and Animal Model

In these studies, we used the human colon carcinoma cell line HT-29 (HTB-38; American Type Culture Collection, Manassas, VA, USA). The cells were incubated in a humidified atmosphere with 5% CO_2_ at 37°C. Dulbecco’s modified Eagle’s medium (DMEM 11995-065; Invitrogen, Carlsbad, CA, USA), supplemented with 10% fetal bovine serum and antibiotics, was used for cell culture. Exponentially growing cells were used in the study. The cells were detached from the plates with trypsin.

BALB/c male nude mice (6 weeks old, 20–25 g body weight) were obtained from Japan SLC (Hamamatsu, Japan). Before the experiments, the mice were undisturbed for at least 1 week. HT-29 cells suspended in phosphate-buffered saline (1×10^7^ cells) were subcutaneously injected into the shoulders of the BALB/c nude mice. The biodistribution and PET studies were performed 3 weeks after the tumor cell implantations. HT-29 tumor-bearing mice were fasted for more than 16 h before the administration of ^64^Cu-ATSM.

### In vivo Biodistribution

For the biodistribution study, a tracer dose of ^64^Cu-ATSM (185 kBq/mouse) was injected into the mice. First, prior to the experiment with penicillamine, the 24-h biodistribution and excretion of ^64^Cu-ATSM were determined in detail in non-tumor bearing BALB/c nude mice. For the penicillamine study, the biodistribution of ^64^Cu-ATSM in nude mice with HT-29 tumor treated with penicillamine p.o. (150 µL in volume), as clinically prescribed, was examined and compared with that in mice treated with saline (control). In the first step, the appropriate injection interval between ^64^Cu-ATSM and penicillamine was determined. Penicillamine (500 mg/kg) was administered at 10 min before, 10 min after, or 1 h after ^64^Cu-ATSM injection, and the biodistribution in these animals was compared. Next, to determine the adequate penicillamine injection dose, the following treatment conditions for single-dose and fractionated administration were compared. To evaluate single-dose administration, 100, 300, or 500 mg/kg of penicillamine were administered at 1 h after ^64^Cu-ATSM injection. To evaluate fractionated administration, 3 doses of penicillamine were administered at 100 mg/kg per dose; the doses were given at 1- or 2-h intervals, starting at 1 h after ^64^Cu-ATSM injection (administration at 1, 2, and 3 h or 1, 3, and 5 h after ^64^Cu-ATSM injection). For laxative treatment, 3 mL of a Glycerin Enema Solution (Yoshida Pharmaceutical, Tokyo, Japan) was administered rectally, according to the manufacturer’s protocol, at 30 min after the fractionated penicillamine dosage schedule of 1, 3, and 5 h after ^64^Cu-ATSM injection. Biodistribution was studied in 4 mice per group at 5 min, 15 min, 30 min, 1 h, 2 h, 4 h, and 6 h and 3 mice per group at 16 h and 24 h after ^64^Cu-ATSM injection. Animals in the 5-min to 6-h sampling groups were housed individually, and the urine and feces were collected with polyethylene-laminated filter paper. Animals in the 16-h and 24-h sampling groups were housed individually in metabolic cages (3600M021; Tecniplast S.p.A, Buguggiate, Italy) to collect urine and feces. The organs of interest (liver, kidney, small intestine, large intestine, muscle, tumor, and remainder of the body) and blood were also collected and weighed. Radioactivity levels were counted with a γ-counter (1480 Automatic gamma counter Wizard 3; PerkinElmer Inc., Waltham, MA, USA). The biodistribution data were calculated and shown as the %ID/g for the organs and blood and the %ID for the urine and feces.

### Small-animal PET Imaging with ^64^Cu-ATSM

The effect of penicillamine on a therapeutic dose of ^64^Cu-ATSM in HT-29 tumor-bearing mice was examined with PET imaging. In this study, ^64^Cu-ATSM (37 MBq/mouse) was intravenously injected, followed by a single-dose p.o. administration of 300 mg/kg penicillamine or saline (control) at 1 h after ^64^Cu-ATSM injection (*n = *3/group). At 0.5, 2, 3, 4, 5, 6, 7, 8, and 24 h after the ^64^Cu-ATSM injection, 5-min emission scans were performed with a small animal PET system (Inveon; Siemens Medical Solutions, Malvern, PA, USA) while the mice remained under 1.5–2% isoflurane anesthesia. Body temperature was maintained with a heat pump during the scans. The images were reconstructed using a 3D maximum a posteriori with Inveon Acquisition Workplace software (Siemens Medical Solutions). Regions of interest (ROIs) were manually drawn over the tumors and livers, and tracer uptake was quantified as the mean standard uptake value (SUV_mean_) in each ROI measured with ASIPro software (CTI Molecular Imaging/Siemens) as described previously [29], [30].

### Dosimetry Analysis

Mean absorbed doses of ^64^Cu-ATSM (mSv/MBq) in humans were estimated on the basis of the biodistribution data that were obtained as described above from HT-29 tumor-bearing mice. The mean %ID/g values of the mouse liver, kidney, small intestine, large intestine, and remainder of the body were converted into corresponding human values according to the standard body weights for mice (20 g) and humans (73.7 kg) [31]. These values were processed with OLINDA/EXM software [32], which used a dynamic bladder model with a voiding interval of 4.8 h to estimate the organ doses (mSv/MBq).

### Statistical Analysis

Data are expressed as the means ± SD. *P* values were calculated using the two-sided *t*-test for comparisons between 2 groups or analysis of variance followed by post-hoc Tukey’s test for multiple group comparisons. *P* values of <0.05 were considered to be statistically significant.

## Results

### Penicillamine-induced Depletion of ^64^Cu from ^64^Cu-ATSM in Mouse Plasma

First, the ability of penicillamine to deplete ^64^Cu from ^64^Cu-ATSM was examined in vitro with mouse plasma (Figure S1). ^64^Cu-ATSM was stable in the plasma in the absence of penicillamine. The relative ratio of intact ^64^Cu-ATSM activity to total activity decreased immediately after the addition of penicillamine, whereas the ratio of the activity of the origin fraction with the free ^64^Cu and ^64^Cu-penicillamine increased. This indicated that trans-chelation from ^64^Cu-ATSM to penicillamine occurred in the plasma and that hydrophilic ^64^Cu-complexes such as ^64^Cu-penicillamine remained at the origin during normal-phase TLC.

### Biodistribution and Excretion of ^64^Cu-ATSM in Mice

**Figure 1:**
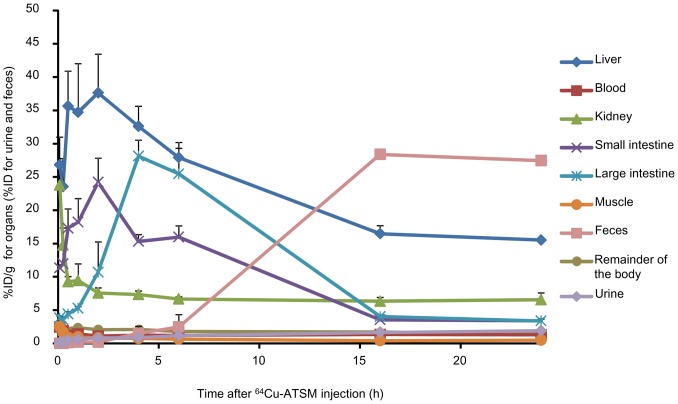
Biodistribution of ^64^Cu-ATSM in BALB/c nude mice. Data were obtained at 5^64^Cu-ATSM injection. Values are expressed as the %ID/g for the organs (liver, kidney, small intestine, large intestine, muscle, and remainder of the body) and blood and as the %ID for the urine and feces. Values are shown as means ± SD; *n* = 4 for 5 min, 15 min, 30 min, 1 h, 2 h, 4 h, and 6 h and *n* = 3 for 16 h and 24 h.

Next, the 24-h biodistribution and excretion of ^64^Cu-ATSM was examined in tumor-free BALB/c nude mice after i.v. injections of ^64^Cu-ATSM; this was conducted because there were no sufficient data describing the distribution and excretion of ^64^Cu-ATSM in mice, particularly at later time points (e.g., 16 h and 24 h) [2], [6], [21], [28]. Time-activity curves for the collected organs and urinary and fecal excretion are shown in Figure 1. Noticeable ^64^Cu accumulation was observed in the liver, small intestine, and large intestine during the first 6 h after injection. Large amounts of ^64^Cu were excreted in the feces by 16 hours after the injection, but little urinary excretion was observed in mice.

### In vivo Biodistribution with Penicillamine Administration

**Figure 2:**
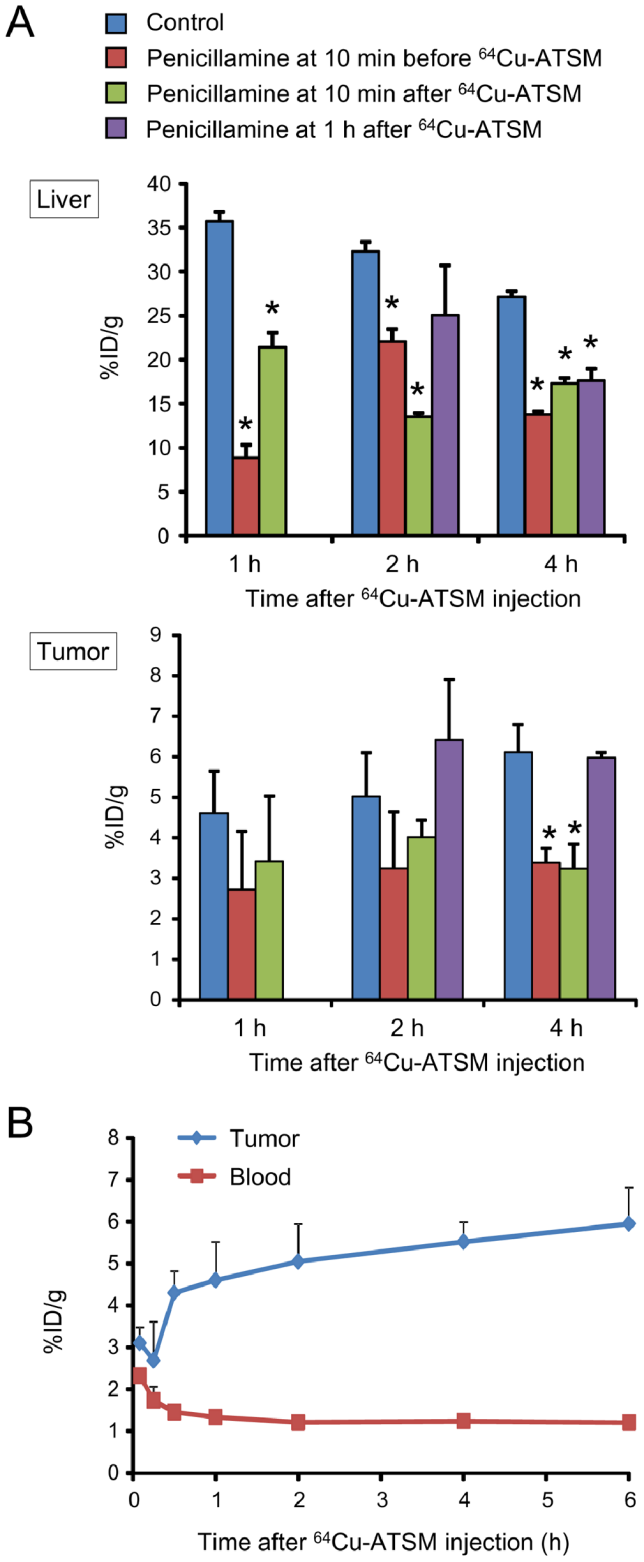
The effect of penicillamine administration timing on the biodistribution of ^64^Cu-ATSM. (A) Biodistribution in the liver (upper) and tumor (lower) in HT-29 tumor-bearing mice that were treated p.o. with a single-dose of 500 mg/kg penicillamine at 10 min before or after ^64^Cu-ATSM injection or at 1 h after ^64^Cu-ATSM injection. Data were obtained at 1, 2, and 4 h after ^64^Cu-ATSM injection. (B) Time activity curves for the tumors and blood, based on the biodistribution data from HT-29 tumor-bearing mice (control animal). Values are shown as means ± SD; *n* = 4 for each time point.

The biodistribution of ^64^Cu-ATSM was compared between various administration schedules in HT-29 tumor-bearing mice that received various p.o. penicillamine doses. First, we examined the biodistribution in animals that were treated with penicillamine (500 mg/kg) at 10 min before, 10 min after, or 1 h after the ^64^Cu-ATSM injection (Figure 2A). In this experiment, the penicillamine dose was set at 500 mg/kg, as this was the maximum concentration that could be dissolved in the injection volume; also, this dose was shown to be below the LD50 in mice that received a single-dose oral injection (720 mg/kg, Material Safety Data Sheet of penicillamine, CAS#52-67-5). Penicillamine treatments at 10 min before or 10 min after ^64^Cu-ATSM injection significantly reduced the ^64^Cu accumulation in the liver (*P*<0.05); however, tumor uptake was also significantly reduced (*P*<0.05). In contrast, treatment with penicillamine at 1 h after ^64^Cu-ATSM injection significantly reduced the liver accumulation (*P*<0.05), whereas there were no significant decreases in tumor accumulation. The time activity curves for the blood and tumors of HT-29 tumor-bearing mice without penicillamine treatment showed that the radioactivity was mostly cleared from the blood and tumor uptake had plateaued by 1 h (Figure 2B).

**Figure 3:**
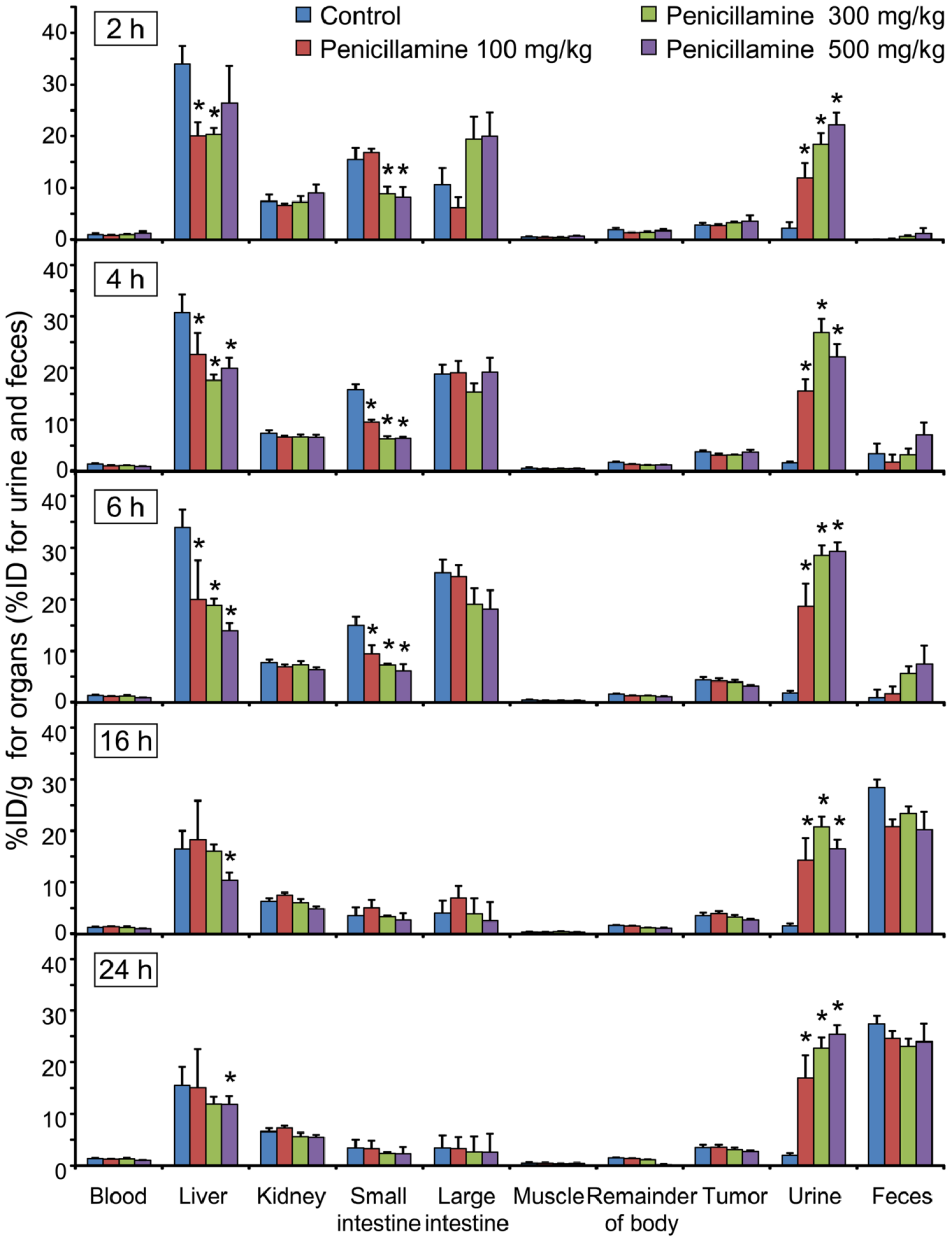
Chronological changes in biodistribution and excretion after ^64^Cu-ATSM injection with single-dose penicillamine injections. Biodistribution in the organs and urinary and fecal excretions at 2, 4, 6, 16, and 24^64^Cu-ATSM injection in HT-29 tumor-bearing mice that were treated p.o. with a single-dose of 100, 300, or 500 mg/kg penicillamine at 1 h after ^64^Cu-ATSM injection, compared with those in animals treated with saline (control). Data are shown as the %ID/g for the organs (liver, kidney, small intestine, large intestine, muscle, remainder of the body, and tumor) and blood and the %ID for the urine and feces. Asterisks indicate statistical significance (*P<0.05) in comparison to the control at each time point. There were no significant differences in the %ID/g of the tumors between any of the treatment groups and the control. Values are shown as means ± SD; *n* = 4 for 2, 4, and 6 h and *n* = 3 for 16 and 24 h.

**Figure 4:**
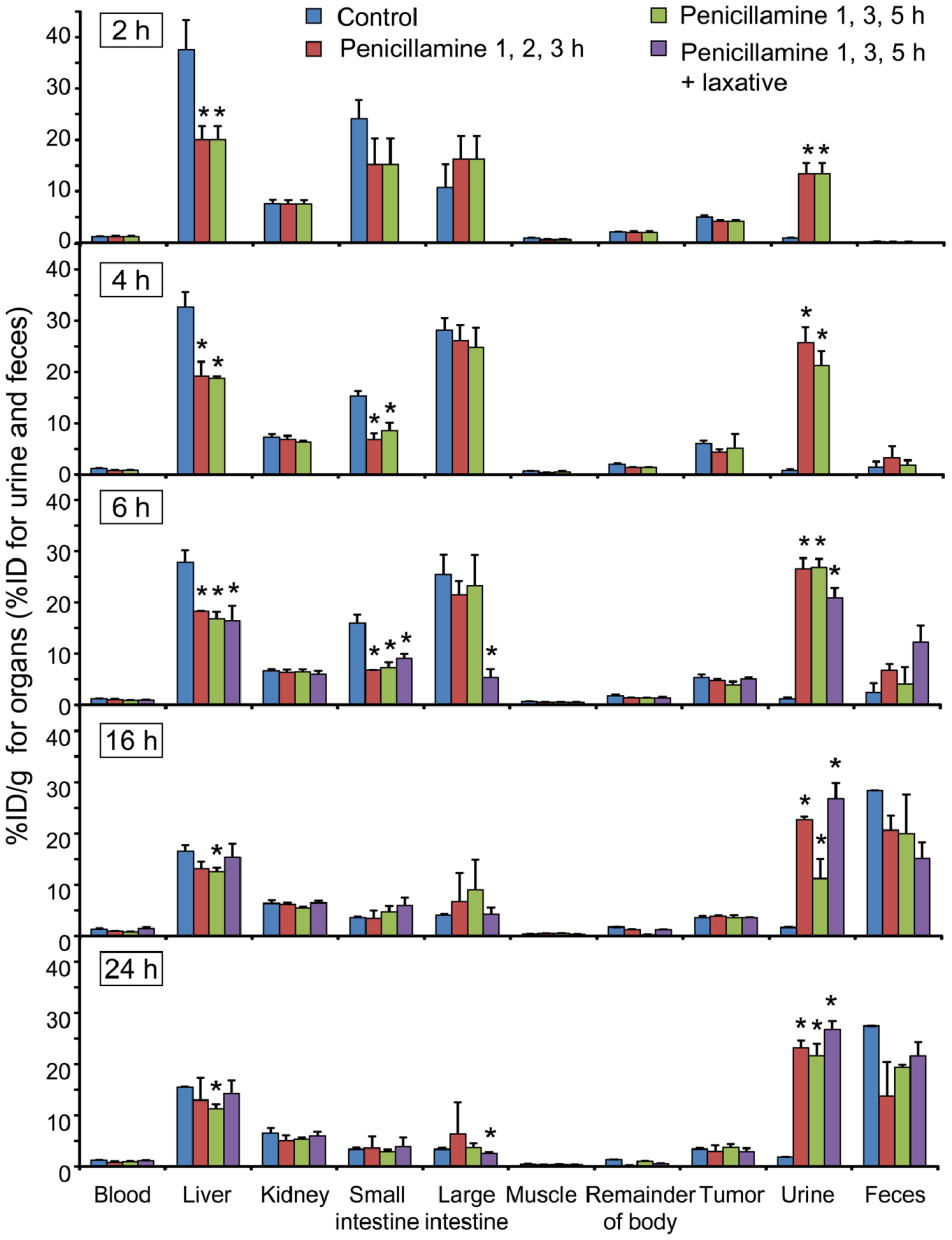
Chronological changes in biodistribution and excretion after ^64^Cu-ATSM injection with fractionated penicillamine injections. Biodistribution in organs and urinary and fecal excretions at 2, 4, 6, 16, and 24^64^Cu-ATSM injection in HT-29 tumor-bearing mice that were treated p.o. with fractionated doses of penicillamine (100 mg/kg×3) at 1, 2, and 3 h or 1, 3, and 5 h after ^64^Cu-ATSM injection, compared with those in animals treated with saline (control). Additional laxative treatments at 5.5 h after ^64^Cu-ATSM injection along with penicillamine administration (100 mg/kg×3; 1, 3, and 5 h after ^64^Cu-ATSM injection) are also shown. Data are shown as the %ID/g for the organs (liver, kidney, small intestine, large intestine, muscle, remainder of the body, and tumor) and blood and the %ID for the urine and feces at 2, 4, 6, 16, and 24 h after ^64^Cu-ATSM injection. Asterisks indicate statistical significance (*P<0.05) in comparison to the control at each time point. There were no significant differences in the %ID/g of the tumors between any of the treatment groups and the control. Values are shown as means ± SD; *n* = 4 for 2, 4, and 6 h and *n* = 3 for 16 and 24 h.

To determine the adequate penicillamine injection dose, various single-dose and fractionated administration conditions were compared in detailed biodistribution studies (Figures 3, 4). For a better understanding, time-activity curves were generated from the biodistribution data for the selected organs with relatively high ^64^Cu accumulation (i.e., liver, small intestine, and large intestine; Figure S2). Initially, we tested single-dose p.o. injections of 100, 300, and 500 mg/kg of penicillamine in HT-29 tumor-bearing mice at 1 h after the ^64^Cu-ATSM injections. Chronological changes in biodistribution in the collected organs and urinary and fecal excretion for the 24-h period after ^64^Cu-ATSM injections with single-dose administrations of penicillamine are shown in Figure 3 (time-activity curves, Figure S2A). These demonstrated that all of the penicillamine treatment groups showed significant decreases in ^64^Cu accumulation in the liver and small intestine, compared to the control (*P*<0.05). Figure 3 also indicates statistical significance in comparison to the control at each time point and shows significant decreases in ^64^Cu accumulation in the liver and small intestine at 4 and 6 h in all treatment groups. In contrast, there were no significant differences in tumor uptake between any of the treatment groups in single-dose administration and the control. The penicillamine treatment significantly accelerated the urinary excretion of ^64^Cu, but increased ^64^Cu retention was not observed in the kidneys. Additionally, the 300 mg/kg penicillamine group showed significantly reduced^ 64^Cu accumulation in the liver and small intestine in comparison to that of the 100 mg/kg dose group (*P*<0.05), while there was no significant difference between the 300 and 500 mg/kg dose groups. Among the doses tested in this study, 300 mg/kg was sufficient for single-dose administration of penicillamine at 1 h after ^64^Cu-ATSM injection.

Next, we examined the effects of fractionated administration in order to maintain a constant effective penicillamine concentration in the blood for a longer time, as well as to lower the dose of penicillamine given in each injection. Administration regimens that comprised an initial 100 mg/kg dose of penicillamine at 1 h after ^64^Cu-ATSM injection followed by 2 additional 100 mg/kg doses at either 1-h or 2-h intervals were compared (penicillamine administration at 1, 2, and 3 h or 1, 3, and 5 h after ^64^Cu-ATSM injection). Chronological changes in biodistribution and excretion for the 24-h period after the ^64^Cu-ATSM injections while examining the fractionated administration of penicillamine are shown in Figure 4 (time-activity curves, Figure S2B). At earlier time points by 6 h, 3 penicillamine doses of 100 mg/kg per dose reduced ^64^Cu accumulation in the liver and small intestine to nearly the same extent as a single-dose injection of 300 mg/kg (Figure 4 compared to Figure 3). When penicillamine administration was fractionated at 2-h intervals after ^64^Cu-ATSM injection, significant decreases in liver accumulation were observed when compared to the control group at later time points (16 and 24 h; *P*<0.05); however, this was not true for the 300 mg/kg single-dose administration or fractionated administration at 1-h intervals (Figures 3, 4). Mice that received fractionated penicillamine administration did not show significant decreases in tumor uptake when compared to the controls. Overall, the biodistribution study demonstrated that the fractionated administration of penicillamine (100 mg/kg×3) at 1, 3, and 5 h after ^64^Cu-ATSM injection was the most favorable of the treatment protocols examined in this study.

### Effect of Penicillamine on a Therapeutic ^64^Cu-ATSM Dose

**Figure 5:**
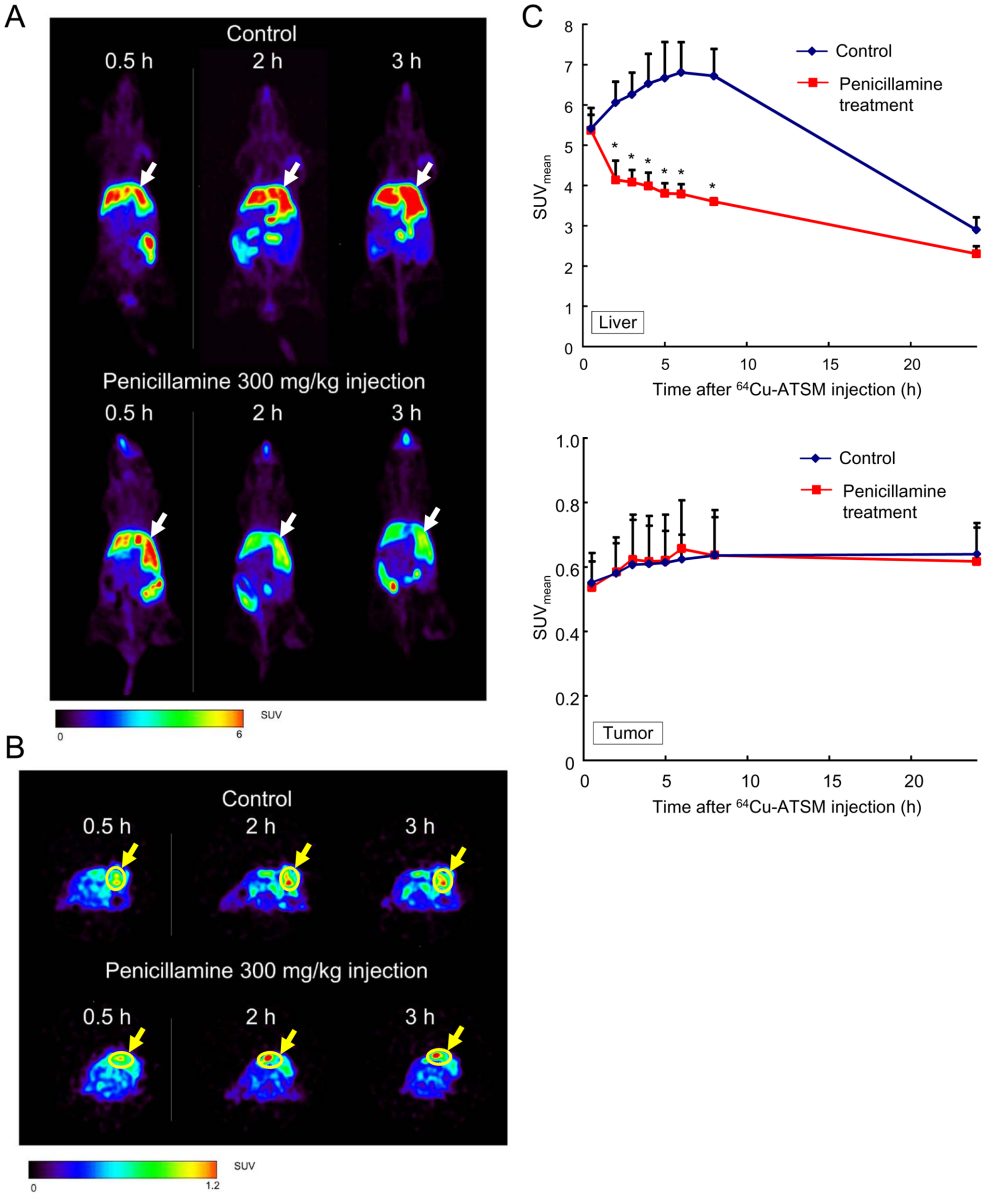
Representative PET images. PET images of HT-29 tumor-bearing mice that were administered ^64^Cu-ATSM and a single dose of penicillamine (300 mg/kg p.o.) or saline (control) at 1 h after the ^64^Cu-ATSM injection are shown. (A) Coronal PET images show the liver (white arrows). (B) Transverse PET images show the tumor. HT29 tumors are indicated by yellow arrows and circles. Images were obtained at 0.5, 2, and 3 h after the ^64^Cu-ATSM injection. (C) Time activity curves from the PET analysis. Time activity curves for the liver (upper) and tumor (lower) are shown for animals treated with penicillamine or saline (control; *n = *3/group). Values are shown as means ± SD.

The biodistribution study demonstrated the effect of penicillamine on the tracer dose (185 kBq/mouse) of ^64^Cu-ATSM. Next, small animal PET with ^64^Cu-ATSM was performed to examine the effect of penicillamine on a therapeutic dose of ^64^Cu-ATSM (37 MBq/mouse), which has been reported to induce therapeutic effects in mice [16], [17] and is equivalent to the therapeutic dose required to increase the survival times of tumor-bearing hamsters [1]. In the PET study, a single-dose p.o. injection of 300 mg/kg at 1 h after ^64^Cu-ATSM injection was employed, rather than fractionated administration, as it allowed us to simply analyze serial changes in ^64^Cu accumulation after a penicillamine administration. Representative whole-body coronal and transverse PET images and time-activity curves from the image analysis are shown in Figure 5. Similar to the results of the biodistribution study, ^64^Cu accumulation in the liver was significantly reduced by penicillamine treatment (*P*<0.05), while tumor uptake was not affected. This indicates that penicillamine functioned similarly with both the therapeutic and tracer doses of ^64^Cu-ATSM.

### Co-administration of a Laxative with Penicillamine

Among the organs with relatively high ^64^Cu accumulation after ^64^Cu-ATSM administration, penicillamine administration reduced ^64^Cu accumulation in the liver and small intestine, but not in the large intestine. Therefore, we performed an additional experiment to determine whether a laxative could reduce ^64^Cu accumulation in the large intestine. For this experiment, we used animals that were treated with a fractionated dose of penicillamine (100 mg/kg per dose at 1, 3, and 5 h) after ^64^Cu-ATSM injection. Prior to the experiment, we confirmed that a glycerin enema significantly increased defecation (*P*<0.05; Figure S3). Anatomical examinations showed that the feces were almost completely evacuated from the large intestine (data not shown). Biodistribution data from the animals treated with both penicillamine and laxative are shown in the latter 3 panels of Figure 4 (time-activity curves, Figure 2B). At 6 h after the ^64^Cu-ATSM injection (30 min after laxative injection), ^64^Cu accumulation in the large intestine was reduced to 20.9% of the control level (*P*<0.05), and the amount of excreted ^64^Cu was increased in the fecal matter (Figure 4). Biodistribution in the other organs was almost similar to that in animals treated with only penicillamine (100 mg/kg×3 at 1, 3, and 5 h after ^64^Cu-ATSM injection), although the significant reductions in liver accumulation at the later time points (16 and 24 h) was not observed in animals treated with penicillamine and laxative (Figure 4). These data indicated that the co-administration of a laxative with penicillamine was effective for quickly reducing ^64^Cu accumulation in the large intestine, although the prolonged effect of penicillamine was slightly weakened.

### Dosimetry Analysis

The mean absorbed doses of ^64^Cu-ATSM (mSv/MBq) in humans were estimated according to biodistribution data from mice that were treated with saline (control) or penicillamine, either via single-dose (300 mg/kg at 1 h after ^64^Cu-ATSM injection) or fractionated (100 mg/kg each at 1, 3, and 5 h after ^64^Cu-ATSM injection with/without laxative) administration (Table 1). The dosimetry data indicate that the liver showed the highest absorbed doses during ^64^Cu-ATSM IRT, with estimated absorbed doses to the liver of 0.108 mSv/MBq in the absence of penicillamine (control) and 0.082 and 0.069 mSv/MBq (24% and 36% reductions from control levels) with single-dose and fractionated penicillamine administration, respectively. Similarly, reduced absorbed doses were seen in the other organs in the penicillamine-treated groups, except for the urinary bladder wall. The accelerated urinary excretion of the radioactive material in penicillamine-treated groups contributed to increased absorbed doses to the urinary bladder wall. The co-administration of a laxative with penicillamine showed a largely reduced radiation dose to the large intestines (46% from control), although reductions in the doses to other organs were slightly lessened (Table 1).

**Table 1:** Mean estimated human absorbed doses for ^64^Cu-ATSM, extrapolated from mouse biodistribution data.

	Estimated absorbed dose (mSv/MBq)			
Target Organ	Control	Penicillamine300 mg/kg	Penicillamine100 mg/kg 1, 3, 5 h	Penicillamine 100 mg/kg 1, 3, 5 h+laxative
Adrenals	0.014	0.011	0.008	0.009
Brain	0.008	0.006	0.004	0.005
Breasts	0.009	0.007	0.004	0.005
Gallbladder wall	0.020	0.015	0.012	0.013
Lower large intestinal wall	0.062	0.043	0.056	0.033
Small intestine	0.053	0.037	0.039	0.044
Stomach wall	0.012	0.009	0.006	0.007
Upper large intestinal wall	0.061	0.042	0.055	0.033
Heart wall	0.011	0.009	0.006	0.007
Kidneys	0.031	0.029	0.025	0.027
Liver	0.108	0.082	0.069	0.078
Lungs	0.011	0.008	0.006	0.007
Muscle	0.010	0.008	0.005	0.006
Ovaries	0.014	0.011	0.009	0.009
Pancreas	0.014	0.011	0.008	0.009
Red marrow	0.009	0.007	0.005	0.006
Osteogenic cells	0.018	0.015	0.009	0.011
Skin	0.008	0.006	0.004	0.005
Spleen	0.010	0.008	0.006	0.007
Testes	0.009	0.007	0.005	0.006
Thymus	0.009	0.007	0.005	0.006
Thyroid	0.009	0.007	0.004	0.005
Urinary bladder wall	0.012	0.069	0.045	0.067
Uterus	0.012	0.011	0.008	0.010
Total body	0.013	0.010	0.008	0.009

## Discussion

^64^Cu-ATSM IRT is a promising cancer treatment that targets over-reduced hypoxic regions of tumors, which are related to malignant characteristics, and is expected to be used in clinical applications [1], [3], [16], [17], [18]. However, high physiological ^64^Cu accumulation in non-target organs is an issue that affects the clinical application of this agent. In particular, the liver is reported to be the principal dose-limiting organ in human ^64^Cu-ATSM PET [21]. In this study, we showed for the first time that during ^64^Cu-ATSM IRT, the appropriately scheduled administration of a copper chelator, penicillamine, could reduce radiation doses to critical organs without decreasing the dose to the tumor. These findings indicate that our new method will provide an additional benefit to ^64^Cu-ATSM IRT by protecting critical organs from radiation damage while maintaining the anti-tumor therapeutic effects.

In this study, we showed that the appropriately scheduled administration of penicillamine could reduce ^64^Cu accumulation in both the liver and the small intestine. Penicillamine has been reported to bind Cu in the small intestine, leading to excretion through the urine, which prevents the enterohepatic circulation of Cu and reduces the accumulation of Cu in the liver [24]. Additionally, orally administered penicillamine was reportedly transported to the liver [26], which could explain how penicillamine might clear accumulated Cu in the liver. Upon consideration of the pharmacokinetics and pharmacodynamics of penicillamine, the substantial reductions of ^64^Cu accumulation in the liver and small intestine were reasonable. Our findings suggest that in ^64^Cu-ATSM IRT, both of the action mechanisms of penicillamine play important roles in reducing the radiation dose to the liver. Furthermore, this study demonstrated that penicillamine administration increased urinary ^64^Cu excretion, although ^64^Cu activity was scarcely retained in the kidney. Therefore, as shown in the present study, penicillamine can transchelate ^64^Cu from ^64^Cu-ATSM and possibly also from free or protein-bound ^64^Cu, and subsequently, ^64^Cu-penicillamine is quickly excreted from the kidney into urine, which adds the benefit of facilitating the clearance of ^64^Cu from the body.

Our biodistribution findings indicated that penicillamine accelerated the renal clearance of ^64^Cu relative to the control. As a result, the estimated absorbed dose to the urinary bladder wall was increased by penicillamine administration (Table 1). We used the dynamic bladder model in the OLINDA/EXM software with a voiding interval of 4.8 h to estimate the absorbed dose. Therefore, it would be beneficial to hydrate patients during an initial period of several hours after ^64^Cu-ATSM administration to reduce the voiding interval. In such cases, the radiation dose from the urinary bladder to critical organs such as the ovaries could be reduced by more than half in cases with voiding intervals of 2 h or less.

According to the biodistribution data, we showed that the fractionated administration of penicillamine at 2-h intervals (100 mg/kg each at 1, 3, and 5 h after ^64^Cu-ATSM injection) was the most favorable of the examined treatment conditions. This is because of the ability of the fractionated dose, given at 2-h intervals, to decrease ^64^Cu accumulation in the liver over a prolonged period. Based on the estimated human absorbed ^64^Cu-ATSM doses (mSv/MBq) in this study (Table 1), the fractionated administration of penicillamine at 2-h intervals showed a 36% reduction in the absorbed dose to the liver relative to the control. Previous therapeutic studies of ^64^Cu-ATSM IRT in hamsters showed that a single administration of 370 MBq ^64^Cu-ATSM effectively increased the experimental survival time of tumor-bearing hamsters without serious toxicity. Based on these results, the authors concluded that the sufficient therapeutic dose of ^64^Cu-ATSM in humans would be 278 GBq [1]. In this study, to anticipate the effects of ^64^Cu-ATSM IRT on non-target organs, we attempted to estimate the radiation doses from an assumed ^64^Cu-ATSM injection dose of 278 GBq/man (Table S1) and to compare these estimates with reported tolerance doses [33], [34]. We found that in the absence of penicillamine, the estimated radiation doses to the liver, ovaries, and red marrow reached or exceeded the tolerance doses (Table S2). Therefore, radiation doses to these organs should be carefully considered as dose-limiting factors in ^64^Cu-ATSM IRT. This study also showed that the fractionated administration of penicillamine could reduce the radiation doses to these critical organs below the tolerance doses (Table S2). In the small and large intestines, radiation doses in the absence of penicillamine were estimated to be below the tolerance doses with an assumed injection dose of 278 GBq/man ^64^Cu-ATSM (Table S2). This suggests that the intestines are not critical organs in ^64^Cu-ATSM IRT, although the ^64^Cu accumulation levels were relatively high. Nonetheless, supportive penicillamine or penicillamine/laxative treatments would help to reduce unnecessary radiation doses to the small and large intestines, respectively. In clinical settings, laxative use might be recommended only if the patients are expected to experience high radiation doses in the large intestine due to colorectal dysfunction or other disorders.

Furthermore, in this study, we found that penicillamine treatment at 1 h after ^64^Cu-ATSM injection, when the blood radioactivity level was already low and tumor uptake had plateaued, could reduce liver uptake without decreasing tumor uptake. On the other hand, penicillamine treatment at 10 min before or after ^64^Cu-ATSM injection reduced tumor uptake. This is likely because penicillamine depleted ^64^Cu from the circulating ^64^Cu-ATSM. These data indicate that the initial penicillamine dose should be administered after the blood radioactivity level has been adequately reduced and the tumor uptake has plateaued. In a clinical study with radiolabeled Cu-ATSM, the blood radioactivity level reportedly decreased and the tumor uptake achieved steady-state at approximately 20 min after injection [35]. Together, these data suggest that in humans, the adequate timing for penicillamine administration after ^64^Cu-ATSM injection might be earlier than in mice. PET guidance would be helpful not only for determining the therapeutic ^64^Cu-ATSM dose, but also for optimizing the penicillamine dose and administration schedule to improve patient outcomes in response to ^64^Cu-ATSM IRT.

Our study showed that the single-dose administration of 300 mg/kg penicillamine or the fractionated administration of 100 mg/kg×3 doses was appropriate for reducing radiation exposure to critical organs during ^64^Cu-ATSM IRT. Animal tests with penicillamine have also demonstrated that the LD50 in mice for single-dose administration is 720 mg/kg (Material Safety Data Sheet of penicillamine, CAS#52-67-5) and that doses less than 400 mg/kg/day did not induce any adverse effects [36]. These findings suggest that the penicillamine dose used in this study was sufficiently low, compared with the toxic levels, and could be safely applied to humans, although the human doses of penicillamine in combination with ^64^Cu-ATSM IRT must be carefully investigated in future clinical studies.

In recent years, due to the recognition of the usefulness of ^64^Cu and its improved availability, drug development and human translational studies with ^64^Cu-labeled compounds have been accelerated. For example, ^64^Cu-chelator conjugates such as ^64^Cu-TETA-octreotide and ^64^Cu-DOTA-trastuzumab have been used in clinical studies [37], [38]. It is noted that ^64^Cu-chelator conjugates often cause ^64^Cu accumulation in the liver due to the hepatic clearance and transchelation of ^64^Cu [39], [40], [41]. In the present study, we developed a novel strategy to remove accumulated ^64^Cu in the liver after ^64^Cu-ATSM injection. This method might be also applied to remove transchelated ^64^Cu in the liver subsequent to other ^64^Cu-labeled compounds. Further studies would be necessary to expand the applicability of this method in the future.

## Conclusions

This study revealed that appropriately scheduled penicillamine administration could reduce radiation to critical organs during ^64^Cu-ATSM IRT without reducing radiation to the tumor. This method therefore promotes the clinical application of ^64^Cu-ATSM IRT.

## Supporting Information

Figure S1 **Ability of penicillamine to remove ^64^Cu from ^64^Cu-ATSM.** Incubation of ^64^Cu-ATSM in plasma with or without penicillamine. The y-axis shows the relative ratios of the fraction with intact ^64^Cu-ATSM and of the fraction at the origin with free ^64^Cu and ^64^Cu-penicillamine. Values are means ± SD, *n* = 3.
(TIF)

Figure S2 **Time-activity curves for liver, small intestine, and large intestine.** Time-activity curves were generated using biodistribution data in Figure 3 and Figure 4. (A) Single-dose administration of penicillamine. (B) Fractionated-dose administration of penicillamine and co-administration of a laxative with penicillamine. Statistical significance at each time point is shown in Figure 3 and Figure 4.
(TIF)

Figure S3 **Quantification of defecation after glycerin enema in mice.** Values indicate the weight of collected fecal matter during a 30-min period after glycerin treatment or from untreated control mice. *P<0.05. Values are means ± SD, *n* = 3.
(TIF)

Table S1 (DOCX)

Table S2 (DOCX)
