# Supplementary figures and images for: Controlled Administration of Penicillamine Reduces Radiation Exposure in Critical Organs during 64Cu-ATSM Internal Radiotherapy: A Novel Strategy for Liver Protection
Source: PLoS One. 2014 Jan 22;9(1):e86996. doi: 10.1371/journal.pone.0086996 (PMC3899369; doi:10.1371/journal.pone.0086996)

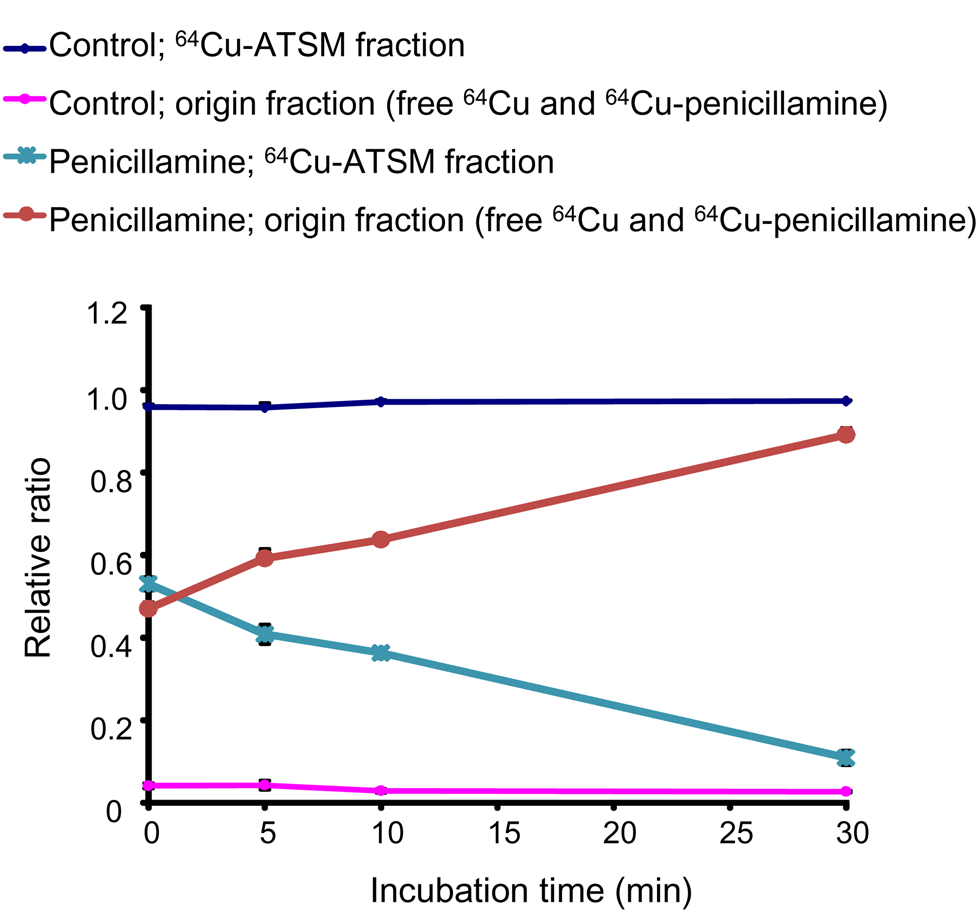

Supplement: Figure S1 — Ability of penicillamine to remove 64Cu from 64Cu-ATSM. Incubation of 64Cu-ATSM in plasma with or without penicillamine. The y-axis shows the relative ratios of the fraction with intact 64Cu-ATSM and of the fraction at the origin with free 64Cu and 64Cu-penicillamine. Values are means ± SD, n = 3. (TIF) [file pone.0086996.s001.tif]

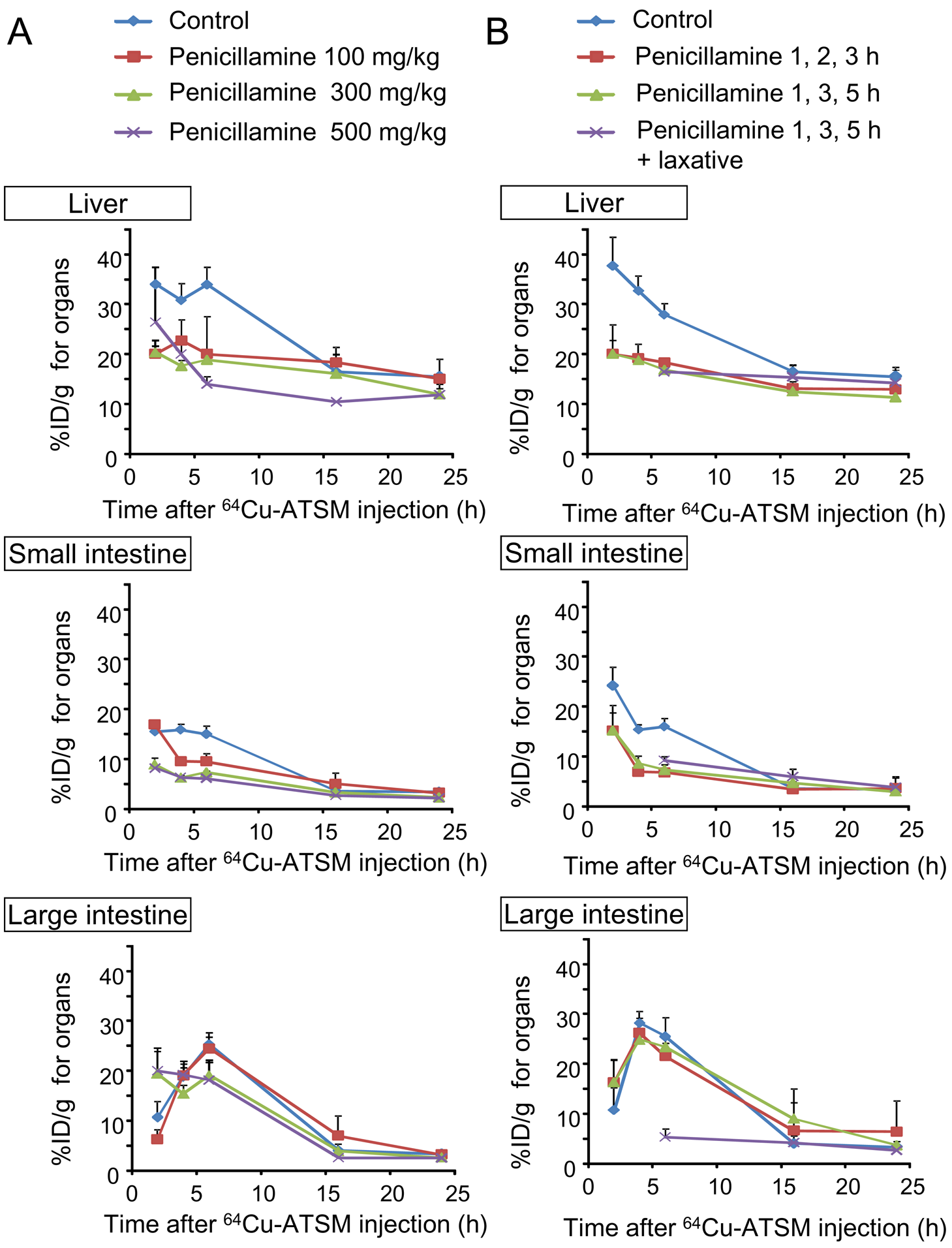

Supplement: Figure S2 — Time-activity curves for liver, small intestine, and large intestine. Time-activity curves were generated using biodistribution data in Figure 3 and Figure 4. (A) Single-dose administration of penicillamine. (B) Fractionated-dose administration of penicillamine and co-administration of a laxative with penicillamine. Statistical significance at each time point is shown in Figure 3 and Figure 4. (TIF) [file pone.0086996.s002.tif]

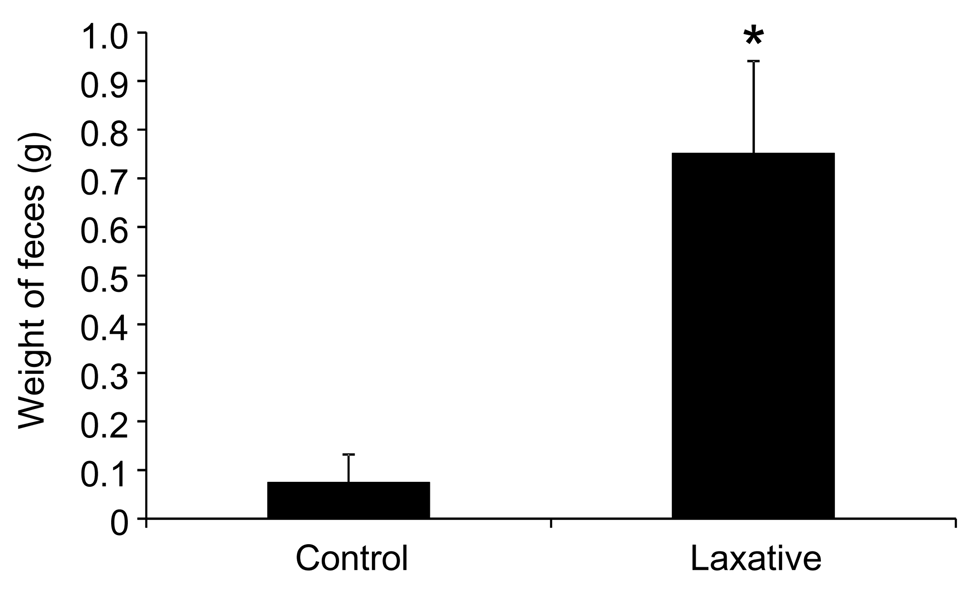

Supplement: Figure S3 — Quantification of defecation after glycerin enema in mice. Values indicate the weight of collected fecal matter during a 30-min period after glycerin treatment or from untreated control mice. *P<0.05. Values are means ± SD, n = 3. (TIF) [file pone.0086996.s003.tif]
