# Supplementary material for: Controlled Administration of Penicillamine Reduces Radiation Exposure in Critical Organs during 64Cu-ATSM Internal Radiotherapy: A Novel Strategy for Liver Protection
Source: PLoS One. 2014 Jan 22;9(1):e86996. doi: 10.1371/journal.pone.0086996 (PMC3899369; doi:10.1371/journal.pone.0086996)
Supplement: Table S1 — (DOCX) [file pone.0086996.s004.docx]

**Supporting Information**

Title: Controlled administration of penicillamine reduces radiation exposure in critical organs during ^64^Cu-ATSM internal radiotherapy: a novel strategy for liver protection

Journal: *PLOS ONE*

Authors: Yukie Yoshii, Hiroki Matsumoto, Mitsuyoshi Yoshimoto, Takako Furukawa, Yukie Morokoshi, Chizuru Sogawa, Ming-Rong Zhang, Hidekatsu Wakizaka, Hiroshi Yoshii, Yasuhisa Fujibayashi, and Tsuneo Saga

Corresponding author: Yukie Yoshii, Molecular Imaging Center, National Institute of Radiological Sciences, Anagawa, Chiba 263-8555, Japan. Phone: +81 43-206-3429; Fax: +81 43-206-0818; E-mail: yukiey@nirs.go.jp.

| **Table S1.** | | | | |
| --- | --- | --- | --- | --- |
| Estimated dose in humans following administration of 278 GBq ^64^Cu-ATSM | | | | |
|  | Estimated dose in human (Sv/278 GBq)^a^ | | | |
| Target Organ | Control | Penicillamine 300 mg/kg | Penicillamine 100 mg/kg 1, 3, 5 h | Penicillamine 100 mg/kg 1, 3, 5 h + laxative |
| Adrenals | 3.86 | 3.03 | 2.17 | 2.55 |
| Brain | 2.22 | 1.75 | 1.11 | 1.35 |
| Breasts | 2.37 | 1.87 | 1.23 | 1.48 |
| Gallbladder wall | 5.42 | 4.17 | 3.28 | 3.67 |
| Lower large intestinal wall | 17.32 | 11.98 | 15.65 | 9.15 |
| Small intestine | 14.76 | 10.15 | 10.84 | 12.34 |
| Stomach wall | 3.22 | 2.52 | 1.80 | 2.06 |
| Upper large intestinal wall | 17.04 | 11.76 | 15.21 | 9.20 |
| Heart wall | 3.17 | 2.49 | 1.69 | 2.02 |
| Kidneys | 8.67 | 8.03 | 6.98 | 7.45 |
| Liver | 30.02 | 22.82 | 19.04 | 21.68 |
| Lungs | 2.92 | 2.28 | 1.54 | 1.85 |
| Muscle | 2.69 | 2.16 | 1.47 | 1.73 |
| Ovaries | 3.81 | 3.06 | 2.47 | 2.61 |
| Pancreas | 3.81 | 2.97 | 2.13 | 2.49 |
| Red marrow | 2.50 | 1.98 | 1.40 | 1.62 |
| Osteogenic cells | 5.12 | 4.03 | 2.63 | 3.17 |
| Skin | 2.23 | 1.77 | 1.16 | 1.40 |
| Spleen | 2.86 | 2.27 | 1.54 | 1.82 |
| Testes | 2.41 | 2.04 | 1.33 | 1.61 |
| Thymus | 2.59 | 2.04 | 1.33 | 1.60 |
| Thyroid | 2.44 | 1.93 | 1.23 | 1.49 |
| Urinary bladder wall | 3.28 | 19.18 | 12.59 | 18.68 |
| Uterus | 3.42 | 3.06 | 2.27 | 2.66 |
| Total body | 3.64 | 2.86 | 2.11 | 2.43 |

^a^The values were calculated based on the dosimetry analysis shown in Table 1.
