# Supplementary material for: Controlled Administration of Penicillamine Reduces Radiation Exposure in Critical Organs during 64Cu-ATSM Internal Radiotherapy: A Novel Strategy for Liver Protection
Source: PLoS One. 2014 Jan 22;9(1):e86996. doi: 10.1371/journal.pone.0086996 (PMC3899369; doi:10.1371/journal.pone.0086996)
Supplement: Table S2 — (DOCX) [file pone.0086996.s005.docx]

**Supporting Information**

Title: Controlled administration of penicillamine reduces radiation exposure in critical organs during ^64^Cu-ATSM internal radiotherapy: a novel strategy for liver protection

Journal: *PLOS ONE*

Authors: Yukie Yoshii, Hiroki Matsumoto, Mitsuyoshi Yoshimoto, Takako Furukawa, Yukie Morokoshi, Chizuru Sogawa, Ming-Rong Zhang, Hidekatsu Wakizaka, Hiroshi Yoshii, Yasuhisa Fujibayashi, and Tsuneo Saga

Corresponding author: Yukie Yoshii, Molecular Imaging Center, National Institute of Radiological Sciences, Anagawa, Chiba 263-8555, Japan. Phone: +81 43-206-3429; Fax: +81 43-206-0818; E-mail: yukiey@nirs.go.jp.

| **Table S2.** | | | | | | |
| --- | --- | --- | --- | --- | --- | --- |
| Comparison between estimated and tolerance doses in critical organs during ^64^Cu-ATSM IRT | | | | | | |
|  |  |  | Estimated absorbed dose [mSv/MBq] | | | |
|  |  |  | *Estimated dose in organ [Sv/278 GBq]*^c^ | | | |
| Organ^a^ | Tolerance dose of radiation [Sv]^b^ | Incidents | Control | Penicillamine 300 mg/kg | Penicillamine 100 mg/kg 1, 3, 5 h | Penicillamine 100 mg/kg 1, 3, 5 h + laxative |
| Liver | 30 | Hepatitis and hepatic vein thrombosis | 0.108 | 0.082 | 0.069 | 0.078 |
|  |  |  | *30.02* | *22.82* | *19.04* | *21.68* |
| Ovaries | 3 | Permanent sterility | 0.014 | 0.011 | 0.009 | 0.009 |
|  |  |  | *3.81* | *3.06* | *2.47* | *2.61* |
| Red marrow | 2.5 | Aplasia | 0.009 | 0.007 | 0.005 | 0.006 |
|  |  |  | *2.50* | *1.98* | *1.40* | *1.62* |
| Intestines | 45 | Stenosis and perforation |  |  |  |  |
| Small intestine |  |  | 0.053 | 0.037 | 0.039 | 0.044 |
|  |  |  | *14.76* | *10.15* | *10.84* | *12.34* |
| Lower large intestine |  |  | 0.062 | 0.043 | 0.056 | 0.033 |
|  |  |  | *17.32* | *11.98* | *15.65* | *9.15* |
| Upper large intestine |  |  | 0.061 | 0.042 | 0.055 | 0.033 |
|  |  |  | *17.04* | *11.76* | *15.21* | *9.20* |

^a^Organs considered in ^64^Cu-ATSM IRT are listed. ^b^Ref [*33*], [*34*]. ^c^Estimated dose in organ following administration of 278 GBq during ^64^Cu-ATSM IRT (italic). The values are compared to the tolerance doses of radiation in the text.
